# Optical Limiting Response of Porous Carbon Dispersions

**DOI:** 10.3390/nano14060533

**Published:** 2024-03-17

**Authors:** Bo Gao, Xuhui Zhao, Lihe Yan, Lijiao Yang, Yue Zhang, Tao Lin, Jinhai Si

**Affiliations:** 1 Department of Electronic Engineering, Xi’an University of Technology, Xi’an 710048, China; 2210320011@stu.xaut.edu.cn (X.Z.); 2210320015@stu.xaut.edu.cn (L.Y.); zhangyue@xaut.edu.cn (Y.Z.); lintao@xaut.edu.cn (T.L.); 2 Laboratory of Photonics Technology for Information, School of Electronic Science and Engineering, Xi’an Jiaotong University, No. 28, Xianning West Road, Xi’an 710049, China; liheyan@mail.xjtu.edu.cn (L.Y.); jinhaisi@mail.xjtu.edu.cn (J.S.)

**Keywords:** super large specific surface area, porous carbon, optical limiting, nonlinear scattering

## Abstract

With the wide application of intense lasers, the protection of human eyes and detectors from laser damage is becoming more and more strict. In this paper, we study the nonlinear optical limiting (OL) properties of porous carbon with a super large specific surface area (2.9 × 10^3^ m^2^/g) using the nanosecond Z-scan technique. Compared to the traditional OL material C_60_, the porous carbon material shows an excellent broadband limiting effect, and the limiting thresholds correspond to 0.11 J/cm^2^ for 532 nm and 0.25 J/cm^2^ for 1064 nm pulses, respectively. The nonlinear scattering experiments showed that the OL behavior was mainly attributed to the nonlinear scattering effect, which is caused by the rapid growth and expansion of bubbles in the dispersion induced by laser irradiation, and the scattered light distribution is consistent with the results of Mie’s scattering. These results suggest that porous carbon materials are expected to be applied to the field of laser protection in the future to further protect the human eye and precision optical instruments.

## 1. Introduction

With the wide application of laser application systems in scientific research activities and military weapons, the research on precise optical instruments and human eye protection technology has become increasingly important [1]. As a very important nonlinear optical phenomenon, the optical limiting (OL) effect can be used to protect sensitive optoelectronic devices and eyes [2,3,4]. The ideal OL material possesses the characteristics of high linear transmittance, low limiting threshold, high damage threshold, fast response speed, and wide-response spectral range. In past decades, many organic materials have been extensively studied as good candidates for optical limiters, including fullerene C_60_, porphyrins, and phthalocyanine [5,6,7]. However, as the OL properties of those organic materials mainly originate from their excited state absorption effect, the OL effect can only take place in a certain wavelength range determined by the excited-state energy level of the molecules.

In recent years, carbon-based nanomaterials, including carbon black suspensions [8], carbon dots (CD) [9], carbon nanotubes (CNTs) [10,11,12,13], and graphene [14,15] have attracted much attention as OL materials. It has been demonstrated that the OL effect of carbon-based materials originates mainly from the thermal-induced nonlinear scattering effect, and the limiting range covers wavelengths from the visible to the near-infrared region [16]. In the nonlinear scattering effect, carbon materials can absorb incident laser light and convert it into heat effectively due to their excellent absorption property. The thermal energy is then transferred into the solvent, causing a rapid increase in the temperature and resulting in the formation of solvent bubbles. The formation and rapid expansion of these micro-bubbles could cause strong light scattering and an OL effect in the dispersions. In this process, the transfer of the heat from the carbon material to the solvent depends, to some extent, on the surface area of the material; those with a larger specific surface area could be beneficial for heat transfer from the carbon material to the solvent and, therefore, the OL performance. As newly emerged carbon materials, porous carbon materials have the characteristics of a high specific surface area, strong adsorption capacity, and high thermal stability. Although porous carbon has been used for adsorption, catalysis, electrochemical energy storage, and other fields [17,18,19], the nonlinear optics and OL properties of porous carbon have not been reported.

In this paper, we studied the OL effect and mechanism of porous carbon with a super-large specific surface area using the nanosecond laser open-aperture Z-scan method. The porous carbon material showed a lower limiting threshold and more excellent broadband limiting characteristics compared to the reference sample of C_60_ toluene solution. Nonlinear scattering measurements were performed to expound the OL mechanism of the material, and the results illustrate that the OL behavior may have originated from the nonlinear scattering caused by the growth and rapid expansion of bubbles in the solvent. Moreover, the distribution of scattering light is consistent with the results of Mie’s scattering.

## 2. Experiment and Sample Preparation

In this work, porous carbon materials with a super-large specific surface area were purchased from XFNano Materials Tech Co, Ltd. (Nanjing, China). First, the purchased porous carbon material was dissolved in deionized water by stirring for 30 min. The products were collected by centrifugation. The as-prepared materials were vacuum-dried in an oven at 180 °C and ground to obtain a carbon powder. The dispersions were prepared by adding 5 mg of the porous carbon powder to 10 mL of solvent (10 mL each of ethanol and dimethyl sulfoxide (DMSO)) and were sonicated for 1 h at room temperature with a power of 200 W. All dispersions were stirred separately using a magnetic stirrer for 30 min to prevent agglomeration. All dispersions used in the experiments were transferred to 1 mm thick quartz cuvettes, and all linear transmittances in the test were adjusted to 75%. The well-stirred solution was transferred into a quartz cuvette, and the absorption spectrum of the samples was analyzed using a UV-2600, Shimadzu, Hong Kong, China. Raman spectroscopy was performed using a Raman spectrometer laser confocal microscope (inVia Qontor, Renishaw, Stonehouse, UK) with an excitation wavelength of 532 nm and a test power of 10 mW. The morphology of the porous carbon materials was investigated by field emission scanning electron microscopy (SEM) characterization using a GeminiSEM 500 microscope (Carl Zeiss AG, Oberkochen, Germany) and transmission electron microscopy (TEM) using a JEOL JEM-2100Plus (China Educational Instrument & Equipment Corp, Beijing, China). To analyze the specific surface area and pore size distribution of the porous carbon materials, the Brunauer-Emmett-Teller (BET) method was used with the fully automatic rapid surface and porosity analyzer (BELSORP-Max, MicrotracBEL, Osaka, Japan). To analyze the chemical composition of the porous carbon materials, the X-ray photoelectron spectroscope (XPS) method was used with the Thermo Fisher ESCALAB Xi+, Langrun International, Xi’an, China.

**Figure 1:**
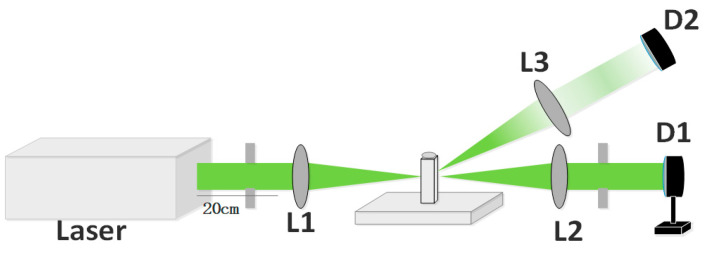
The optical path diagram of aperture Z-scan experiment.

In this experiment, the OL properties of the disordered porous carbon materials with a super-large specific surface area were studied using a nanosecond aperture Z-scan system. As shown in Figure 1, the nanosecond laser source was the Nd^3+^: YAG laser produced by the Continuum Company, Milpitas, CA, USA, with a 10 Hz repetition rate, a 1064 nm center wavelength, and a 10 ns pulse width. After passing through the internal frequency doubling crystal, 532 nm laser pulses were obtained. The output laser pulses were focused on the sample by a lens (L1) with a focal length of 20 cm, and the transmitted light passing through the sample was collimated with L2 and passed through an aperture to filter the scattered light. The sample was fixed on the translation stage and, with changes in the moving position, the nonlinear transmittance of the sample at different positions was obtained by an energy meter (D1). To study the mechanism of OL behavior, part of the scattered light was collected by a convex lens (L3), with a positive angle of about 15° to the beam axis, and recorded by the photodiode (D2). When conducting the angle-dependent experiment of the nonlinear scattering signal, we fixed the distance between the sample and the photodiode (D2) and measured the changes in the scattered light intensity by rotating the angle continuously. 

## 3. Results and Discussion

### 3.1. Characterization Results and Analysis

**Figure 2:**
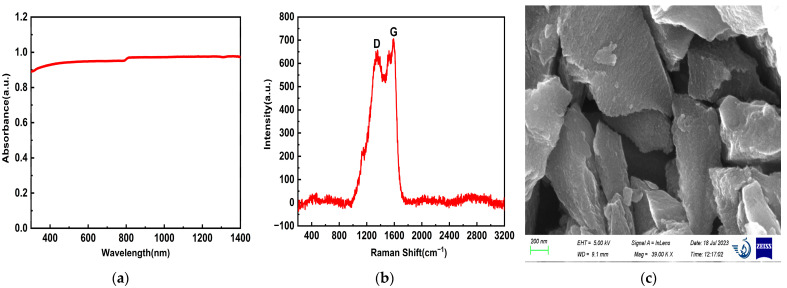

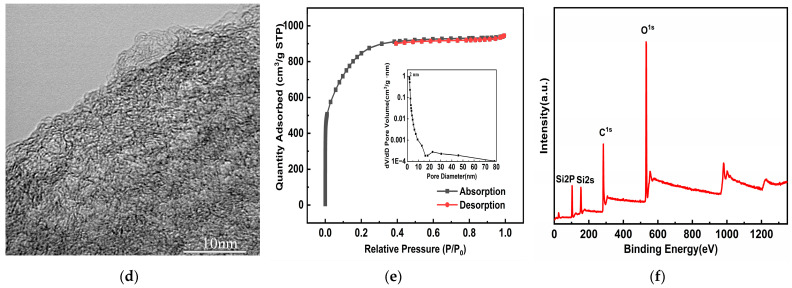
The typical characterization diagrams of porous carbon materials: (**a**) UV–Vis absorption spectrum; (**b**) Raman spectrum; (**c**) SEM image; (**d**) TEM image; (**e**) N_2_ adsorption-desorption isotherm, the inset is the pore size distribution; (**f**) XPS spectrum.

**Figure 3:**
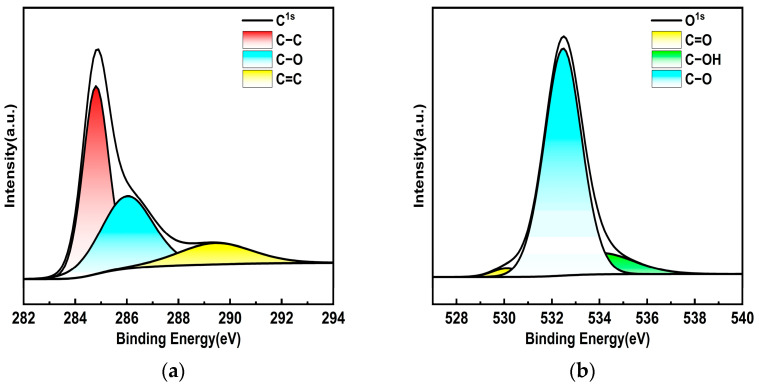
The XPS spectra for porous carbon (**a**) C1s; (**b**) O1s.

In order to analyze the physicochemical properties of porous carbon materials, the morphology and structure of the materials were first characterized. As shown in Figure 2a, the absorption spectrum of porous carbon dispersion was studied using a UV-2600 spectrophotometer in the range of 200–1400 nm. Obviously, the carbon material presented a strong absorption in the visible near-infrared region, showing excellent broadband absorption characteristics. During the test, the same solution was first used for baseline correction, to filter out the impact of the solvent Then, the porous carbon dispersion was transferred to a 1 cm quartz cuvette for measurement. The integrating sphere was added in the measurement process to filter out the influence of scattering. The absorbance of the sample is the absolute absorbance. To study the degree of graphitization of porous carbon materials, Raman characterization was performed. As shown in Figure 2b, the carbon material has the D-characteristic peak at about 1350 cm^−1^, and the G-characteristic peak at 1580 cm^−1^. The G band is caused by the stretching vibration of the SP^2^ atomic pair, which corresponds to the lattice vibration of graphite in the E_2g_ symmetry state; the D band corresponds to the defects and disorder degree of carbon materials [20]. In addition, $I_{D}/I_{G}$ is frequently applied to evaluating carbon material type and graphitization level. Smaller values of $I_{D}/I_{G}$ indicate a higher degree of graphitization of the material. The $I_{D}/I_{G}$ value of porous carbon dispersion is 0.85. The porous carbon material shows the high degree of graphitization and disorder. Defects and disorder in electronic crystals play an important role in the enhancement of the electric field, which can be used to improve the optical limiting effect of devices. The morphology of porous carbon material was observed using SEM as shown in Figure 2c, which exhibited a 100-nanometer-sized structure similar to a “stone” at a magnification of 39,000. In addition, a large number of pores with uneven surface of the material can be seen, which provides support for a high specific surface area. In order to further analyze the surface morphology of porous carbon materials, TEM measurements were carried out. As shown in Figure 2d, the porous carbon material contains a large number of worm-like pore structures, showing a semi-transparent state as a whole, and no particles are observed. In order to understand the pore structure and specific surface area of the materials, the characterization of the nitrogen adsorption isotherm of the porous carbon materials was carried out. According to IUPAC classification, all carbon materials belong to type I/IV isothermal adsorption and desorption curves. The results imply that the specific surface area can reach 2.9 × 10^3^ m^2^/g, which shows the ultra-high specific surface area of the porous carbon material. In addition, the pore volume of the materials is 1.46 cm^3^/g, and the average pore size is 2.02 nm, corresponding to the mesoporous material. As shown in Figure 2e, the adsorption and desorption curve of the carbon material was observed, which shows the mixed adsorption isotherm of type I and type IV [21], indicating the coexistence of micropores and mesopores in the material. The pore size distribution diagram of carbon materials given by the inset of Figure 2e shows that the pores are concentrated at 2 nm, which is consistent with the BET isotherm adsorption results. The large specific surface area and pore size distribution displayed by the carbon material can provide the material with a larger contact and reaction area, improve various physical properties, and have great application value. Porous carbon can effectively shorten the transport path of electrolyte ions and improve the performance of the device by using the synergistic effect of micropores, mesopores, and macropores. In order to analyze the chemical composition of porous carbon materials, XPS spectra were measured. As shown in Figure 2f, the full spectra depicted four typical peaks that correspond to C1s, O1s, Si2p, and Si2s states, which proved the existence of carbon, silicon, and oxygen, corresponding to 284.12 eV, 532.39 eV, 103.2 eV (Si2p), and 154.2 eV (Si2s), with contents of 36.5%, 31.6%, 16.43%, and 15.46%, respectively. In Figure 3a, the high-resolution spectrum C1s could be divided into three peaks, with bonding energies of about 284.4 (C–C), 285.4 (C–O), and 287.9 (C=O). Moreover, the O1s spectra (Figure 3b) reveal that the O1s band can be deconvoluted into three peaks assigned to C=O (530.3 eV), C–O (532.4 eV), and C-OH (534.1 eV) [22]. The Gaussian–Lorentz curve is used to fit the measured spectrum, the area ratio corresponding to each peak is calculated, and the ratio of different covalent bonds in the sample can be obtained. In the C1s spectrum of porous carbon materials, the proportion of C–C bond is 50.98%, the proportion of C–O bond is 36.8%, and the proportion of C=O bond is 12.22%. In the O1s spectrum of porous carbon materials, the proportion of C=O bond is 2.63%, the proportion of C–O bond is 83%, and the proportion of C=O bond is 14.37%.

### 3.2. Characterization Results and Analysis OL Properties and Mechanisms of Porous Carbon Samples

The nonlinear OL behavior of the porous carbon materials for 532 nm and 1064 nm is studied using the nanosecond laser Z-scan technique. We used porous carbon DMSO dispersion for the experiments. During the test, the cuvette was continuously shaken to ensure the stability of the porous carbon dispersion.

**Figure 4:**
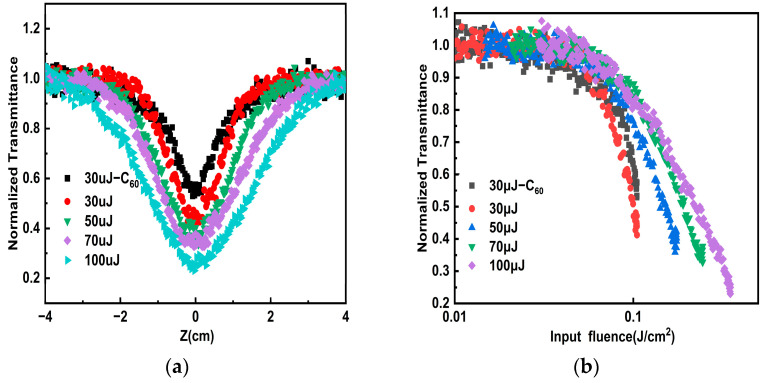
At 532 nm, the contrast curves of porous carbon dispersion and C_60_ toluene solution of (**a**) the normalized transmittance with the z position changes and (**b**) the normalized transmittance with the incident pulse energy density changes.

**Figure 5:**
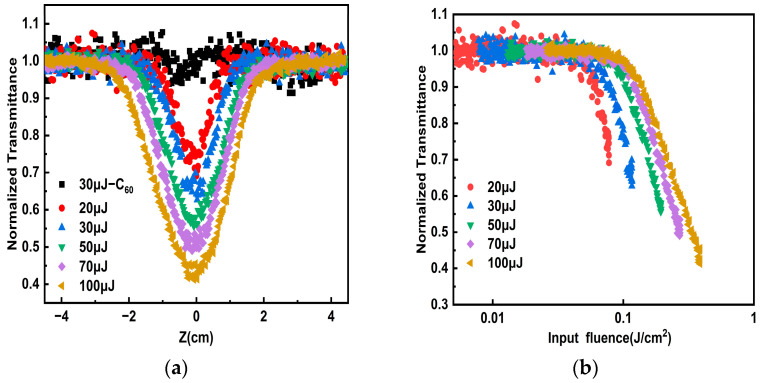
At 1064 nm, the contrast curves of porous carbon dispersion and C_60_ solution of (**a**) the Z-scan curves as the pulse energy changes and (**b**) the transmittance with the incident pulse energy density changes.

In order to study the OL response intensity of the porous carbon material, as a reference, the Z-scan measurements of traditional OL material C_60_ toluene solution were performed. According to the normalized transmittance at position z:

(1) $$T=\frac{T\left(i\right)}{T_{0}},$$

where $T\left(i\right)$ is the transmittance of the sample at different positions, and $T_{0}$ is the linear transmittance of the samples. At 532 nm, keeping the pulse energy from 30 μJ to 100 μJ, the normalized transmittance comparison curves of the porous carbon dispersion and C_60_ toluene solution are shown in Figure 4a. All the samples exhibit the typical reverse saturable absorption (RSA). When the distance from the focus is relatively far, the normalized transmittance of the sample is equal to one, corresponding to the linear regime. The normalized transmittance of the two materials decreases rapidly as the sample approaches the focal position, and the aperture of the transmittance curve of the porous carbon dispersion is larger than the reference sample. And the opening of the transmittance curve of the porous carbon dispersion becomes larger when the pulse energy increases from 30 μJ to 100 μJ. The results indicate that the porous carbon material exhibits the better OL effect at 532 nm. Due to the large specific surface area of porous carbon materials, heat energy can be transferred to surrounding solvents more quickly, showing better RSA. According to the energy density of the incident light at position z:

(2) Iz=I0πω21+z2z02,

we convert the sample position to the energy density of the incident laser. Among them, $I_{0}$ is the incident light energy; $\omega_{0}$ is the beam waist radius of the Gaussian beam at the focal point of 90 μm; $z_{0}$ is the Rayleigh length of 1.5 cm; and z represents the distance from the sample to the focus of the lens (cm). As shown in Figure 4b, the normalized nonlinear transmittance as a function of the incident pulse energy density was measured, of both porous carbon dispersion and C_60_ toluene solution. The limiting threshold is the input flux at which the normalized transmittance of the sample drops to 50% of the linear transmittance. The limiting threshold of porous carbon dispersion was measured as 0.11 J/cm^2^ at 30 μJ at 532 nm, which is lower than the OL threshold of the referenced C_60_ toluene solution (0.13 J/cm^2^). As shown in Table 1, when the pulse energy increases from 30 μJ to 100 μJ, porous carbon precipitate is formed at the bottom of the cuvette, the concentration of porous carbon irradiated by laser decreases, requiring a higher energy density to excite the nonlinear response of the porous carbon material. Then we changed the wavelength of the incident light to 1064 nm to further explore its OL effect in the near-infrared region. As shown in Figure 5a, with the incident power density increases, the normalized transmittance of the porous carbon dispersion decreases rapidly. And the opening of the transmittance curve of porous carbon dispersion becomes larger when the pulse energy increases from 20 μJ to 100 μJ, indicating that the limiting effect is more pronounced. Figure 5b gives normalized transmittance as functions of the incident pulse energy density of the porous carbon dispersion from 20 μJ to 100 μJ at 1064 nm. As shown in Table 2, the porous carbon material has a higher limiting threshold at a higher pulse energy. The normalized transmittance of porous carbon materials at 532 nm is consistent with the change trend of pulse energy density. Therefore, porous carbon materials possess the advantages of low OL threshold and wide response spectrum compared with traditional OL materials, and can be considered as candidate carbon materials for application in the military and medical laser protection [23]. We also compared the optical limiting ability of porous carbon with other materials, as shown in Table 3. The OL ability of porous carbon dispersion in dimethyl sulfoxide has a lower limiting threshold. Compared with N-CD-Pt, rGO-TiO_2_, Au-graphene nanocomposites, CNT’s, indium phthalocyanine/SWCNTs, MWCNTs/TiO_2_, and CNH, the porous carbon materials exhibit a more intentional OL effect.

In previous research, the OL effect of carbon materials mainly comes from nonlinear absorption or nonlinear scattering [13]. For example, L. Vivien et al. indicated that the OL mechanism of carbon nanotubes is mainly sourced from strong nonlinear scattering, which is caused by the growth of solvent bubbles and the sublimation of nanotubes. According to the large specific surface area of porous carbon, the heat transfer efficiency from the particles to the solvent is higher. To clarify the mechanism of the OL effect of porous carbon with a super-large specific surface area, we placed a photodiode at an angle of about 15° with the incident light in the aperture Z-scan optical path to collect part of the forward scattered light, and measured scattered light intensity changes by varying the pulse energy.

**Figure 6:**
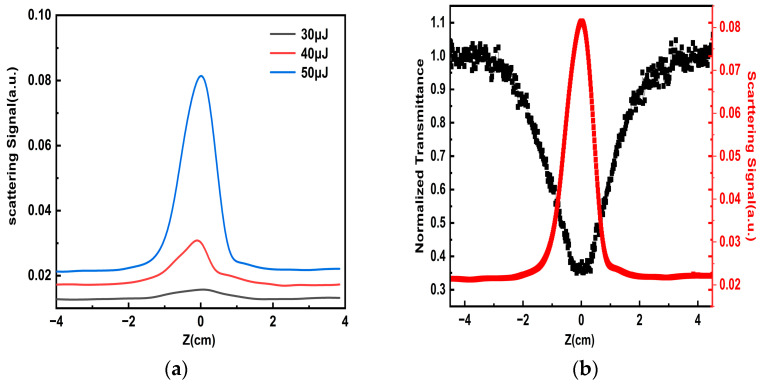
At 532 nm, (**a**) the nonlinear scattering change of porous carbon materials as the pulse energy increases and (**b**) the transmitted and scattered light intensity change in the dependence on the Z position.

We plot the curves of scattering signals at different positions. As shown in Figure 6a, when the porous carbon materials are close to the focus, the intensity of the scattering signal gradually increases. And as the pulse energy increases from 30 μJ to 50 μJ, the scattering light generated by the sample increases. In the linear scattering part, the scattering signal of the porous carbon material is proportional to the pulse energy. As the sample moves closer to the focal point, the nonlinear effect of the porous carbon material is excited and the scattering signal is rapidly enhanced. At 532 nm, the pulse energy is kept at 50 μJ. The curves of nonlinear scattering signals and normalized transmittance are drawn in Figure 6b; with the increase of the incident light power density, the normalized transmittance of the porous carbon dispersion decreases rapidly, and the nonlinear transmittance is lowest at the focus. When the sample is close to the focus, the laser radius changes little, so the measured transmittance changes little. Secondly, there are few data points measured in the center of the focus, and the test accuracy will also lead to saturation.

However, the normalized nonlinear scattering signal of the sample is enhanced synchronously, and the scattering signal is highest at the focal position of the lens, indicating that the OL response of the materials mainly originates from nonlinear scattering. In the OL process of materials, the solvent generates plasma under the action of the laser. The porous carbon dispersion absorbs light from the incident nanosecond laser and converts it into heat energy [30]. Then, the heat energy will be transferred into the solvent, and the positively and negatively charged plasmas are combined to form microbubbles. When the bubble expands to a size comparable to the wavelength, it becomes a scattering center. The time to generate a nonlinear response does not exceed 10 ns, but it cannot be accurate to a specific number. At the scattering center, the strong scattering effect on incident light will be produced by bubbles, which will greatly reduce the transmittance, so as to achieve the OL effect. In addition, the porous carbon material has a large specific surface area, which could achieve the process of transferring the heat energy to dispersion quickly, resulting in the lower limiting threshold and better OL performance.

**Figure 7:**
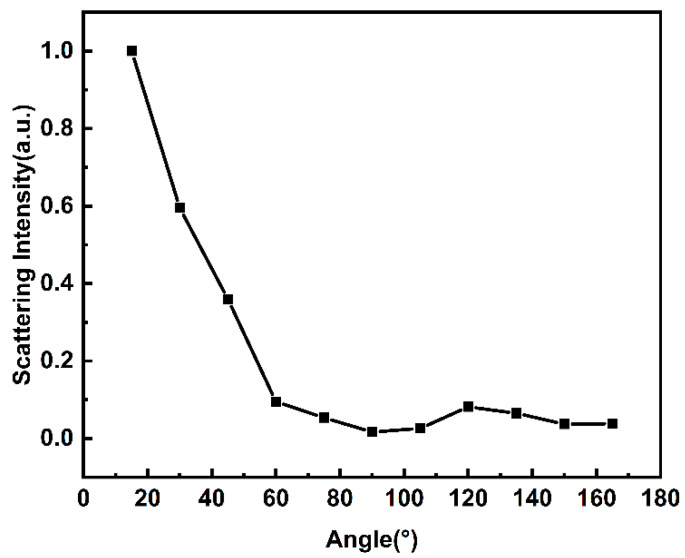
The angle dependence of the normalized scattering intensity of the porous carbon dispersion at 15–165°.

According to Mie’s scattering theory, the distribution of scattering intensity is related to the direction of incident light. To confirm the scattering mode of porous carbon, the angular dependence of the nonlinear scattering signal intensity from 15 to 165° (with a 15° interval) was measured. In the experiments, we have removed the effect of background light and kept the distance between the detector and the sample constant. As shown in Figure 7, the scattered signal intensity changes as the forward angle increases at an incident pulse energy of 50 μJ for 532 nm. From the figure, we can see that the scattered signal intensity decreases when the angle becomes larger, and most of the scattered light is scattered along the forward direction of the incident light (15–90°). Conversely, the backscattered signal intensity is relatively weak. Different from Rayleigh scattering, the directionality of scattering intensity is more obvious in Mie’s scattering [15]. The scattering intensity of the forward direction is stronger than that in the back direction. In the experiment, the results indicate that the scattered light distribution of porous carbon materials conforms to the distribution trend of Mie’s scattering [31]. Therefore, the nonlinear scattering exhibited by this porous carbon material is attributed to the Mie’s scattering generated by the suspension bubbles. These excellent properties shown by porous carbon materials may make them of great value for laser protection in the future.

## 4. Conclusions

In summary, we have investigated the OL mechanism of super-large specific surface area disordered porous carbon (2.9 × 10^3^ m^2^/g) using nanosecond aperture Z-scan technology. The limiting thresholds of porous carbon material and C_60_ are 0.11 J/cm^2^ and 0.13 J/cm^2^ at 30 μJ at 532 nm, respectively. And at 1064 nm, the limiting threshold of porous carbon material is 0.25 J/cm^2^, and C_60_ does not have an optical limiting effect. Porous carbon materials have a lower limiting threshold. Compared with the scattering signal and the normalized transmittance, it is concluded that the OL effect of the carbon material originates from nonlinear scattering, and the large specific surface area makes the heat transfer faster, resulting in more scattering and a lower transmittance rate. At the same time, the nonlinear scattered light distribution is consistent with the Mie’s scattering simulation results. In other words, most of the incident light is scattered along the forward direction. Therefore, the OL performance of porous carbon materials can be further optimized by removing the influence of forward scattered light, such as by placing a diaphragm in front of the detector to block part of the scattered light.

## Figures and Tables

**Table 1:** Comparison of optical limiting properties of porous carbon with different pulse energies at 532 nm.

Sample	Pulse Energy (μJ)	The Minimum Value of Normalized Transmittance	OL Threshold
C_60_	30	51.2%	0.13 J/cm^2^
Porous carbon	30	38.8%	0.11 J/cm^2^
Porous carbon	50	35.7%	0.15 J/cm^2^
Porous carbon	70	32.7%	0.19 J/cm^2^
Porous carbon	100	22.9%	0.22 J/cm^2^

**Table 2:** Comparison of optical limiting properties of porous carbon with different pulse energies at 1064 nm.

Pulse Energy (μJ)	The Minimum Value of Normalized Transmittance	OL Threshold
20	74.7%	
30	66.4%	
50	55.1%	
70	49.8%	0.25 J/cm^2^
100	40.2%	0.32 J/cm^2^

**Table 3:** Comparison of optical limiting properties of several materials.

Sample	Normalized Transmittance at the Focal Point	Optical Limiting Threshold	Reference
Porous carbon at 532 nm	42.7%	0.11 J/cm^2^	This paper
Porous carbon at 1064 nm	49.8%	0.25 J/cm^2^	This paper
N-CD-Pt at 532 nm	30.1%	0.62 J/cm^2^	[11]
rGO-TiO_2_ at 1030 nm	29.7%	1.5 J/cm^2^	[24]
Au-graphene nanocomposites at 532 nm	23.2%	0.4 J/cm^2^	[25]
CNT’s at 532 nm	37.9%	3 J/cm^2^	[26]
indium phthalocyanine/SWCNTs at 532 nm	24.9%	0.21 J/cm^2^	[27]
MWCNTs/TiO_2_ at 532 nm	19%	0.22 J/cm^2^	[28]
CNH at 532 nm	43%	1.8 J/cm^2^	[29]

## Data Availability

The data that support the findings of this study are available from the corresponding author upon reasonable request.
